# Acute Myeloid Leukemia in Patients Living with HIV Infection: Several Questions, Fewer Answers

**DOI:** 10.3390/ijms21031081

**Published:** 2020-02-06

**Authors:** Fabio Forghieri, Vincenzo Nasillo, Francesca Bettelli, Valeria Pioli, Davide Giusti, Andrea Gilioli, Cristina Mussini, Enrico Tagliafico, Tommaso Trenti, Andrea Cossarizza, Rossana Maffei, Patrizia Barozzi, Leonardo Potenza, Roberto Marasca, Franco Narni, Mario Luppi

**Affiliations:** 1 Section of Hematology, Department of Medical and Surgical Sciences, University of Modena and Reggio Emilia, Azienda Ospedaliero-Universitaria di Modena, Policlinico, 41124 Modena, Italy; vincenzo.nasillo@unimore.it (V.N.); francesca.bettelli@unimore.it (F.B.); valeria.pioli@unimore.it (V.P.); davide.giusti@unimore.it (D.G.); andrea.gilioli@unimore.it (A.G.); rossana.maffei@unimore.it (R.M.); patrizia.barozzi@unimore.it (P.B.); leonardo.potenza@unimore.it (L.P.); roberto.marasca@unimore.it (R.M.); franco.narni@unimore.it (F.N.); mario.luppi@unimore.it (M.L.); 2 Section of Infectious Diseases, Department of Surgical, Medical, Dental and Morphological Sciences. University of Modena and Reggio Emilia, Azienda Ospedaliero-Universitaria di Modena, Policlinico, 41124 Modena, Italy; cristina.mussini@unimore.it; 3 Center for Genome Research, Department of Medical and Surgical Sciences, University of Modena and Reggio Emilia, Azienda Ospedaliero-Universitaria di Modena, 41124 Modena, Italy; enrico.tagliafico@unimore.it; 4 Department of Laboratory Medicine and Pathology, Unità Sanitaria Locale, 41124 Modena, Italy; t.trenti@ausl.mo.it; 5 Section of Immunology, Department of Medical and Surgical Sciences, University of Modena and Reggio Emilia, 41124 Modena, Italy; andrea.cossarizza@unimore.it

**Keywords:** acute myeloid leukemia, HIV infection, AIDS, anti-retroviral therapy, acute promyelocytic leukemia, myelodysplastic syndrome, hematopoietic stem cell transplantation

## Abstract

Both human immunodeficiency virus (HIV) infection and acute myeloid leukemia (AML) may be considered relatively uncommon disorders in the general population, but the precise incidence of AML in people living with HIV infection (PLWH) is uncertain. However, life expectancy of newly infected HIV-positive patients receiving anti-retroviral therapy (ART) is gradually increasing, rivaling that of age-matched HIV-negative individuals, so that the occurrence of AML is also expected to progressively increase. Even if HIV is not reported to be directly mutagenic, several indirect leukemogenic mechanisms, mainly based on bone marrow microenvironment disruption, have been proposed. Despite a well-controlled HIV infection under ART should no longer be considered per se a contraindication to intensive chemotherapeutic approaches, including allogeneic hematopoietic stem cell transplantation, in selected fit patients with AML, survival outcomes are still generally unsatisfactory. We discussed several controversial issues about pathogenesis and clinical management of AML in PLWH, but few evidence-based answers may currently be provided, due to the limited number of cases reported in the literature, mainly as case reports or small retrospective case series. Prospective multicenter clinical trials are warranted to more precisely investigate epidemiology and cytogenetic/molecular features of AML in PLWH, but also to standardize and further improve its therapeutic management.

## 1. Introduction

Human immunodeficiency virus (HIV) infection causes disruption of the adaptive immune system through dysfunction and loss of CD4+ T cells [1,2]. Progressive deterioration of host immunity occurs with increased risk of opportunistic infections and malignancy compared with the general population and possible development of acquired immunodeficiency syndrome (AIDS) [2,3]. Furthermore, HIV infection is associated with chronic immune activation and systemic inflammation, concurrently with immunosenescence and T cell exhaustion, which could causally be linked to the increased cancer risk [3,4,5]. In addition to HIV-related immunosuppression, which also impairs the control of oncogenic viral infections, people living with HIV infection (PLWH) are more frequently exposed to cancer risk factors, such as smoking and alcohol abuse, potentially contributing to elevated risk of malignancy [6,7]. Since the introduction of effective anti-retroviral therapy (ART) in late 1995, HIV-related morbidity, the number of newly diagnosed AIDS, AIDS-related deaths, and incidence of AIDS-defining cancers (ADC), including Kaposi Sarcoma, aggressive non-Hodgkin lymphoma, and cervical cancer have dramatically decreased by greater than 70%, with concurrent substantial improvement of survival [7,8,9,10]. However, the incidence of ADC continues to be higher than in the general population, and aging due to longer life expectancy in the ART era has led to increased incidence of non-AIDS-defining malignancies [3,6,11,12,13]. In general, the lifetime risk of developing cancer still remains 25% to 40% in PLWH receiving ART, with malignancies accounting for approximately 33% of all HIV-related deaths [11,14,15,16,17]. The elevation of cancer-related mortality for many malignancy subtypes among HIV-infected patients compared with HIV-uninfected subjects is not only related to advanced tumor stage or differences in treatment approaches, but also potentially reflects a direct correlation between immunosuppression and tumor progression [18]. Acute myeloid leukemia (AML) is considered among non-AIDS-defining hematological malignancies and several challenging topics about its epidemiology, pathogenesis, and clinical outcomes in PLWH will be discussed below.

## 2. Which is the Actual Epidemiology of AML in PLWH?

In 2018, an estimated 37.9 million people worldwide were living with HIV, with a global HIV infection prevalence of 0.8–1% among adult population, mainly aged 15–49 years. The vast majority of PLWH are located in low- and middle-income countries, with an estimated 68% living in sub-Saharan Africa, where 5.2% of the population is considered to be infected. Therefore, although relatively uncommon in Western countries, HIV infection continues to be a major global public health issue [19]. On the other hand, AML is recognized as the most common acute leukemia type in adults, affecting an estimated 0.5% of the general population at some point during lifetime, with a yearly incidence of 3–5 cases per 100,000 individuals and a median age at diagnosis of 68 years [20,21]. Therefore, both HIV infection and AML may be considered as relatively uncommon disorders in the general population, but the precise frequency of AML occurrence in the setting of HIV infection is uncertain, because epidemiological studies are very limited and show some controversial results [22]. Of note, the median life expectancy of PLWH treated with modern ART is actually 72 to 75 years, compared with less than 65 and 55 years observed in 2010 and 2000, respectively [14,23,24]. As mentioned above, these gradual improvements in life expectancy and clinical conditions parallel with a significant increase in absolute incidence of non-AIDS-defining cancers, including non-AIDS-defining hematological malignancies [6,22,25,26,27]. Of interest, HIV serological testing is accounted among the procedures to be carried out in the initial work-up of a newly diagnosed AML patient in either general practice or clinical trial setting [28]. At our Institution, two out of 276 AML patients (0.72%), consecutively observed over a 10-year period between 2009 and 2018, showed HIV positivity at serological analysis [Personal observation]. On the other hand, among the 51 HIV-positive patients from our Institution, who needed to undergo diagnostic BM aspiration and trephine biopsy in the same time period, to investigate for any hematological abnormality, AML was documented in the two above mentioned cases (3.9%), whereas no cases of myelodysplastic syndrome (MDS) were observed [Personal observation]. A 2007 meta-analysis of the incidence of cancer in PLWH found an increased leukemia incidence in HIV-positive subjects, but an association between HIV and specific leukemia subtypes was unfortunately not identified [22,29]. While some previous data from Italian Cancer and AIDS Registries reported a decline in incidence ratio for leukemia in HIV-positive patients [30,31], most evidence from the literature suggests that AML may occur at increased frequency in PLWH, though remaining a relatively infrequent complication [22,32,33]. In brief, a 2.5-fold increase in leukemia frequency has been documented in HIV-infected individuals from the United States [34], while a two-fold increase in AML incidence has been calculated in a French study, compared with the general population [35]. Consistent with this, the estimated incidence of AML in Japanese HIV-positive people was 8/100,000 persons per year, between 1991 and 2009 [36]. A more recent retrospective national multicenter study from France revealed that acute leukemia incidence in PLWH was not significantly different than in general population, but acute leukemia occurred earlier (mean age 50 years for AML patients), compared with HIV-negative counterparts [37]. Kaner et al. also documented that AML in PLWH presented at a younger age, compared with known historical data from HIV-negative patient cohort (median age 56 vs. 69 years) [38]. However, the occurrence of malignancies not typically associated with immunosuppression induced by HIV infection, especially those malignancies characterized by increased incidence with age, such as AML, may progressively become more prevalent as the HIV-infected population ages [39]. Furthermore, immunosuppression is also known to increase the risk of developing AML in the setting of solid organ transplantation, especially in case of intense and prolonged immunosuppression, as administered in heart transplant recipients, further suggesting a potential role of immune surveillance in protecting against myeloid clones outgrowth [40]. In summary, further prospective epidemiological studies are needed to more precisely define the incidence of AML in PLWH [22].

## 3. Are there Distinct Mechanisms of Leukemogenesis Related to HIV Infection?

Before the introduction of ART, anemia, neutropenia, and thrombocytopenia were observed in approximately 70%, 50%, and 40% of AIDS patients, respectively [23,41]. Although the frequency of cytopenias has significantly declined in the ART era, it continues to be a common finding in PLWH, especially in cases of progression from asymptomatic HIV infection to advanced disease and AIDS [41]. Ineffective hematopoiesis from direct suppression of bone marrow (BM) progenitor cells by HIV infection or indirectly through excessive secretion of inflammatory cytokines induced by HIV can blunt hematopoiesis. Moreover, infiltrative BM disease of either neoplastic or infectious origin by opportunistic agents, nutritional deficiencies, autoimmune mechanisms, and adverse effects of medications can pathophysiologically contribute to occurrence of cytopenias, in a multifactorial fashion [41]. It is also well recognized that HIV infection and at least some concurrent anti-retroviral drugs are commonly associated with the development of morphologic changes in BM architecture which mimic MDS. However, mechanisms and outcomes of these HIV-related morphologic myelodysplastic features on BM are not completely understood and it is still unclear whether dysplastic BM changes may confer a higher risk of developing MDS as a distinct disease entity [22,23,42]. Even if HIV infection is not reported to be mutagenic, whether it could per se significantly contribute to the risk to develop either MDS or AML still remains undefined [22] due to previous controversial results on ex vivo experimental models [39]. In detail, Murthy et al. previously isolated HIV from circulating myelomonoblasts in an HIV-positive patient with AML, with concurrent evidence of presumptive HIV replication by the presence of p24 antigen and reverse transcriptase activity in the supernatant of cell culture [43]. Conversely, Farber et al. failed to identify infectious viral particles when donor lymphocytes were co-cultured with leukemic monocytes from an HIV-positive patient affected with AML [44]. Accordingly, Costello et al. also did not observe HIV hybrid fragments in myeloblasts from a HIV-positive subject with monoblastic leukemia, therefore excluding a direct transforming role of HIV on leukemic blasts [45]. On the other hand, it has recently been observed that HIV can affect hematopoietic stem cells (HSCs) in at least a subset of individuals, acting as a reservoir, thereby contributing to viral latency but also potentially leading to their increased susceptibility to apoptosis [41,46,47]. Several additional mechanisms have been potentially attributed to HIV infection in indirectly increasing the predisposition to develop either MDS or AML in PLWH [22,39]. First of all, during acute infection of CD4+ T cells, the trans-activator protein Tat is released extracellularly, therefore playing a major role in BM angiogenesis, which could be relevant for leukemogenesis. Additionally, HIV may disrupt the BM microenvironment, inducing an inflammatory milieu by infecting monocytes and macrophages, with subsequent activation of genes of cytokines, especially G-CSF, GM-CSF or Interleukin-6, putatively involved in development and growth of leukemic stem cells [22,23]. Finally, the basic domain of the above mentioned Tat protein displays the capability to displace preformed basic fibroblast growth factor (bFGF), which is known to increase myelopoiesis directly via FGF receptors on myeloid progenitors [22,39]. It should also be noted that prior exposure to chemotherapy and/or radiotherapy for any previous solid or hematologic malignancy, more frequently lymphomas in PLWH, may significantly facilitate the development of either MDS or AML [22,48]. For instance, therapy-related myeloid neoplasms (t-MN) account for nearly 10%–20% of all MDS or AML cases in the general population, and they could potentially become more frequent in HIV-positive patients, who can actually achieve long-term disease-free survival after having received effective anti-tumor treatment in combination with ART for primary malignancy [22,48]. In addition to these leukemogenic mechanisms, it has long been hypothesized that patients with HIV infection exhibit a global accelerated aging, therefore resulting in development of age-related comorbidities earlier than expected for their age [23,49]. Relevant to this, despite a younger age at presentation, a higher proportion of clonal-hematopoiesis related mutations, mainly *ASXL1* and *DNMT3A* gene mutations, and higher risk cytogenetics have recently been found in HIV-positive MDS individuals, compared with HIV-negative counterparts [23]. However, whether HIV infection, per se, deranged inflammatory BM microenvironment, a direct toxic effect of ART or accentuated aging phenotype could clearly affect hematopoiesis, leading to acquisition of somatic mutations consistent with clonal hematopoiesis of indeterminate potential (CHIP) in PLWH is so far undefined [23]. CHIP is an age-related phenomenon in the general population, with circulating clones detectable in less than 1% of individuals under 40 years of age, but increasing in frequency with each decade of life [50]. Of note, 10% to 20% of individuals aged 70 or older harbor a detectable hematopoietic clone. CHIP has been associated with an increased risk of developing hematologic malignancies, including MDS and AML, at a rate of about 0.5% to 1% per year, but also with cardiovascular diseases and all-cause mortality [50]. To the best of our knowledge, no precise information about prevalence and possible clinical significance of CHIP in PLWH is currently available in the literature, therefore perspective studies are warranted to investigate this challenging issue, with a special focus on dynamics of mutated hematopoietic clones in the context of deficient immune surveillance.

## 4. Should AML in HIV-Positive Subjects Be Managed Differently from Cases Occurring in Patients without HIV Infection?

Since the first case reported in 1986 [51], most data on biological characteristics, clinical presentation, and therapeutic management of AML in PLWH derived from either case reports or small retrospective patient series, as detailed in Table 1 and Table 2. In the series by Sutton et al., conventional intensive AML treatments were administered to 15 of the 18 reported patients and 73% of them achieved complete remission (CR), without HIV-related opportunistic infections, suggesting that HIV infection did not significantly influence short-term AML outcomes, especially in cases with CD4+ T cell counts > 200/µL, good performance status and without pre-existing AIDS diagnosis [35]. However, a high AML relapse rate was finally observed, potentially correlated to difficulties in administering adequately intensive consolidation treatments to HIV-positive patients [35]. Accordingly, despite only a handful of cases were collected in the ART era, most patients reviewed in the manuscript by Aboulafia et al. in 2002 were considered fit to receive induction chemotherapy and 83% of them obtained CR [39]. Even if clinical outcomes were collectively poor, median overall survival (OS) was significantly longer in chemotherapy-treated patients (7.5 months) compared with those who did not receive intensive treatments (1 month). However, relapse commonly occurred, namely in 54% of cases who previously achieved CR [39]. Moreover, patients with a CD4+ T cell count < 200/µL had a dismal median OS of 7 weeks, compared to those cases with a CD4+ T cell count > 200/µL who experienced a median OS of 7 months, suggesting that immune function may show a favorable impact on AML prognosis [39]. More recently, Dy et al. reported 5 newly identified cases and reviewed the 68 previously reported cases until 2011 [52]. Median survival of subjects with CD4+ T cell counts ≥200/µL and < 200/µL was 13.4 and 7 months, respectively. CR was obtained in 33 of 46 intensively treated patients (71.7%), but 51.5% of them relapsed after a median CR duration of 9.2 months. Of interest, median survival of untreated and treated patients was 1.0 and 13.2 months, respectively, with patients who obtained CR showing a median OS of 21 months. According to multivariate analysis, standard AML treatment and reaching CR were associated with longer survival in HIV-positive patients, regardless of CD4+ T cell count [52]. Furthermore, Evans et al. investigated a potential correlation between CD4+ T cell count and cytogenetic features and their role as predictors of survival in a retrospective series accounting 9 newly identified cases and 22 previously published cases for whom analysis of conventional karyotype was available, as detailed in Table 2 [32]. Interestingly, median CD4+ T cell counts at diagnosis were 355/µL, 196/µL, and 60/µL for patients in the favorable, intermediate, and unfavorable cytogenetic risk groups, respectively, even if a statistically significant correlation could not have been established [32]. Median OS for intensively treated patients with favorable and intermediate karyotypes was 10.5 and 13.5 months, respectively, whereas it was 5 months for cases with poor risk cytogenetics, even if statistically significant difference was not reached due to small patient numbers. Of further note, median OS was 8.5 months for intensively-treated patients with favorable and intermediate risk karyotypes and CD4+ T cell count < 200/µL, compared with 48 months for those with CD4+ T cell count ≥200/µL. It could be considered that low CD4+ T cell count may be a strong predictor of survival in HIV-positive AML, regardless of karyotype, therefore offsetting the beneficial prognostic role of favorable karyotype. According to these data, a careful patient stratification based on CD4+ cell counts and karyotype may be suggested, considering induction chemotherapy a reasonable option mainly for HIV-associated AML with CD4+ cells > 200/µL and without unfavorable karyotype [32]. Overall, AML in PLWH has exhibited dismal clinical outcomes, with poorer OS compared with HIV-negative counterparts, despite generally younger age at presentation (Table 1 and Table 2) [32,36,37,38,39,48,53,54]. Scanty information about either cytogenetic or molecular abnormalities is available for cases of AML in PLWH, thereby avoiding the possibility to draw any firm conclusion on their frequencies and prognostic impact (Table 2) [32,36,37,39,48,54]. However, AML could potentially parallel the features of MDS in the setting of HIV-positive patients, which are mainly characterized by high prevalence of complex karyotypes and chromosome 7 abnormalities, resulting in higher risk and faster progression to leukemia, compared to MDS in HIV-uninfected subjects [23,55,56]. Of interest, a complex/unfavorable karyotype was predictive of poor outcome also in acute leukemia and high-risk MDS patients from the recently reported series by Cattaneo et al., whereas no correlation between age, CD4+ T cell count, HIV infection duration, and OS was documented [54]. However, with a median OS of 17 months for the entire cohort, a trend for better OS was observed for patients with acute leukemia or MDS diagnosis in the more recent years (2-year OS 64.2% between 2011 and 2019 compared with 27.3% in the period 1994–2010). This trend of improvement in AML prognosis despite a significantly higher median age at diagnosis recorded in the period 2011–2019, could mainly be attributable to recent advances in approaching AML in PLWH, including better selection of patients candidate to intensive chemotherapy and/or allogeneic hematopoietic stem cell transplantation (alloHSCT), concurrently with careful and prompt management of infectious complications [54]. Consistent with this, a recent French retrospective multicenter study reported that, with a multidisciplinary approach, HIV-positive AML patients could receive intensive chemotherapy, resulting in good efficacy and tolerability [37]. After propensity score matching, no difference in OS (2-year OS 29% for AML and 40% for ALL cases) was observed between PLWH and HIV-negative acute leukemia controls [37]. According to these observations, a well-controlled HIV infection should no longer be considered per se a contraindication to standard intensive chemotherapy for AML treatment and patients inclusion in clinical trials may certainly help to improve and standardize their clinical management [54]. The potential role of either new drugs targeting specific molecular lesions or moderate-intensity treatments based on hypomethylating agents is currently unknown for AML in PLWH and should prospectively be explored in suitable cases.

## 5. Should HIV-Positive Patients Receive ART during Treatment for AML?

HIV infection, especially if untreated, induces impairment of the immune system and BM dysfunction, which can compromise the treatment of AML, by contributing to high rates of life threatening infections, other complications secondary to protracted cytopenias and death, even during consolidation phases [32]. Despite the controversies raised on the specific prognostic significance of CD4+ T cell count on the survival outcomes of HIV-positive AML [32,35,39,52,54], higher response rates and cancer-specific survival are generally observed for HIV-positive cancer patients showing CD4+ T cell count above 200/µL [7,11]. Accordingly, initiation or optimization of ART, with the consultation of an Infectious Diseases specialist, is currently recommended for cancer patients with HIV infection, and treatment with chemotherapy and concurrent ART is increasingly common, even if sometimes hampered by incomplete medication adherence, overlapping toxic effects, suboptimal pharmacokinetics and potential drug-drug interactions [11]. In detail, nucleoside reverse transcriptase inhibitors may be involved in transporter-mediated interactions, while protease inhibitors, non-nucleoside reverse transcriptase inhibitors and chemokine receptor antagonists are extensively metabolized by and variably induce or inhibit CYP450 system. Moreover, pharmacologic boosters are recognized to inhibit CY3A4 [7,11]. For these latter reasons, a few years ago suspension of ART was suggested as the best option during treatment for acute leukemia. Only for cases with scheduled prolonged consolidation and maintenance phases, ART was indicated, with selection of antiretroviral agents guided by the knowledge of pharmacokinetics of various drugs [57]. Conversely, although careful monitoring is needed and modifications to ART may be necessary, continuation of therapy for HIV infection is actually recommended during anti-leukemic treatments, at least to facilitate immune reconstitution, therefore reducing infection mortality [7,14,22]. ART regimens containing integrase inhibitors, such as raltegravir or dolutegravir, without pharmacologic boosters are currently favored in the setting of malignancy, because of their low potential for drug-drug interactions [7,11]. Of note, anthracyclines and antimetabolite agents, which are frequently used for AML treatment, generally undergo non-CYP450 routes of elimination and their metabolism is unlikely to be significantly altered by ART [11]. Given the current availability of ART and evidence of improved immune recovery, it would be unlikely and possibly considered unethical to perform a randomized clinical trial investigating intensively-treated HIV-positive AML patients with or without ART [14]. Indeed, with the widespread use of ART in the last few years and concurrent improvements in prophylactic strategies with agents against bacterial, fungal (including mold-active triazoles) and opportunistic infections, namely mycobacterial infections, *Pneumocystis jirovecii* and herpervirus reactivations, intensive induction, and consolidation therapeutic approaches for AML have become more feasible in PLWH, with an acceptable reduction of infectious morbidity and mortality, even during long-lasting neutropenic phases [7,11,22,58,59,60].

## 6. How should Acute Promyelocytic Leukemia Be Approached in PLWH?

Acute promyelocytic leukemia (APL), characterized by the balanced translocation t(15;17) (q22;q12) resulting in the fusion transcript PML-RARA, is a rare entity, accounting for approximately 10% of AML cases [61]. The widespread use of all-trans retinoic acid (ATRA)-based regimens has significantly revolutionized the treatment of APL, providing a paradigm of molecularly targeted treatment [61]. Furthermore, the chemotherapy-free approach with ATRA in combination with arsenic trioxide (ATO) has been demonstrated to be highly effective in APL and has recently become the standard first-line therapy in non-high risk APL patients, with obtainment of OS rates largely exceeding 90% [62,63,64]. To the best of our knowledge, since 1997 only a few cases of APL have so far been reported among PLWH, as recently reviewed by Kunitomi et al. [65] and summarized in Table 3, thereby avoiding the possibility to give any evidence-based recommendation on therapeutic management of this uncommon clinical entity [65,66]. In the majority of cases, the diagnosis of HIV infection preceded the occurrence of APL by at least two years and most patients received ART, as summarized in Table 3 [35,65,66,67,68,69,70,71,72]. These ten adult patients received remission induction treatment with ATRA, either alone or, more commonly, in combination with chemotherapy, mainly anthracyclines, achieving at least morphologic CR in all but one cases, followed by consolidation/maintenance therapeutic approaches. However, detailed information on obtainment and maintenance of molecular CR is lacking for some patients, as reported in Table 3. Of note, most patients remained alive during the observation period, with last clinical follow-up since APL diagnosis ranging from 8 to 40 months (Table 3) [35,65,66,67,68,69,70,71,72]. Interestingly, in previous in vitro studies, despite ATRA was found to up-regulate HIV mRNA transcription in HIV-infected human HL-60 cell line, no corresponding increase in viral replication was documentable due to a block in HIV mRNA translation and replication, causing the HIV-infected cells to eventually undergo apoptosis [73]. Accordingly, ATRA reduced the proviral DNA load in the HIV-infected 8E5 cell line in a dose-dependent manner [74]. Viral replication was also significantly reduced in primary lymphocytes obtained from 3 HIV-infected patients, suggesting that ATRA could potentially be an effective therapeutic agent even against HIV infection [74]. It should also be noted that in previous in vitro studies, protease inhibitors were found to enhance the ability of ATRA to induce differentiation and inhibit growth of myeloid leukemia cells [66]. On the contrary, earlier studies proposed that ATO may potentially enhance HIV infectivity, mainly through degradation of PML proteins, which are known to be normally recruited in the cytoplasm and to interfere with early steps of viral replication, and through reverse transcriptase stimulation in human T cells [75,76]. However, these previous results have not been fully confirmed in subsequent studies [4]. While ATO has been recognized to potently suppress T cell proliferation, raising concerns for its potential application in PLWH, since CD4+ T cell loss is the major hallmark of HIV infection, some possible antiviral mechanisms have recently also been proposed [4]. ATO has shown efficacy in the traditional “shock and kill” strategy against CD4+ cells through thioredoxin reductase inhibition, with a potential impact on viral reservoir. Moreover, ATO could reduce the susceptibility of subsequent HIV infection down-regulating CCR5 expression on CD4+ T cells [4]. However, a potential clinical application of ATO in PLWH to control HIV infection is currently uncertain. To date, scanty information on the use of ATO in either first-line or salvage treatments for APL in PLWH is available in the literature. Malik et al. in 2009 described the first HIV-infected APL patient, who received ATO as salvage therapy after having experienced relapse and obtained long-lasting second CR [72]. Furthermore, four patients, presumptively affected with APL, from the series by Rabian et al. received a therapeutic approach based on the combination ATRA/ATO, but, unfortunately, no detailed data were provided [37]. Even if definitive conclusions on management of APL in PLWH cannot be drawn, due to the limited number of patients so far described, standard ATRA-based APL therapy combined with ART, preferably including integrase inhibitors in order to reduce potential drug-drug interactions, may be suggested, especially in fit subjects with adequate performance status and well controlled HIV infection, with CD4+ T cell count > 200/µL and absence of history of AIDS-related complications [65,66]. Without currently available data from the literature, the chemo-free combination of ATRA and ATO may be valuable, but should be used with caution in patients with low- and intermediate-risk APL and concurrent HIV infection in the clinical practice, while collection of “real-life” patients’ series and potentially prospective multicenter clinical trials are necessary to investigate this issue [65].

## 7. Is Allogeneic HSCT Feasible and Effective in HIV-Infected Patients Affected with AML?

Since the 1980s, alloHSCT has been suggested as a possible treatment option to eradicate HIV infection, but this strategy was highly unsuccessful, because HIV replication was not affected during conditioning therapy in the absence of antiviral treatment and donor lymphoid cells arising after engraftment were persistently susceptible to HIV infection [77]. Furthermore, in the pre-ART era, the survival outcomes of alloHSCT performed to treat hematological malignancies were poor, mainly due to extremely high infection-related mortality rates [78,79]. After the introduction of ART, leading to advances in HIV management, survival outcomes in PLWH undergoing alloHSCT have significantly improved, resulting similar to those obtained for subjects without HIV infection [78,79,80,81]. Notwithstanding, there still was general reluctance to routinely use alloHSCT in PLWH, mainly because of basal immunosuppression with presumptive high risk of opportunistic infections, complex drug-drug interactions, increase in conditioning regimens-related toxicities, and risk of delayed engraftment, potentially resulting in higher transplant-related mortality (TRM) [80]. However, a recent comprehensive systematic review has shown that alloHSCT outcomes, including engraftment, immune reconstitution, risk of infections, incidence of graft-versus-host-disease (GVHD), other complications and mortality, were globally comparable to those observed in HIV-negative counterparts [80]. Accordingly, the recent phase 2 BMT CTN-0903/AMC-080 multicenter clinical trial prospectively confirmed the safety and effectiveness of alloHSCT, with either myeloablative or reduced-intensity conditioning (RIC) regimens, in PLWH and hematological malignancies, including 9 AML cases [82]. In detail, no cases of non-relapse mortality occurred at 100 days and subsequently at 6 months since alloHSCT. As in the general HIV-negative population receiving alloHSCT, malignancy relapse remained the main cause of treatment failure, with 6-month and 1-year OS were 82.4% and 58.8%, respectively [82]. Although clinical evidence for alloHSCT in PLWH is globally limited, mainly resulting from case reports and small retrospective case series, as recently reviewed elsewhere [80], alloHSCT could actually be a reasonable and potentially curative option for selected patients, with well-controlled HIV infection, who otherwise meet standard criteria for transplant eligibility [79,80,81]. Detailed information so far available from the literature on the use of alloHSCT in HIV-infected patients with AML in the ART era is summarized in Table 4 [36,37,54,81,82,83,84,85,86,87,88,89,90,91,92,93,94,95]. Regarding clinical outcomes, OS for 32 AML patients, collectively analyzed by Arslan et al. [80], was 91.6% and 41.6% at 6-month and 1-year follow-up, respectively. Interestingly, 1-year OS was not significantly different between patients receiving myeloablative conditioning (40%) or non-myeloablative/RIC regimens (42.8%) [80]. These latter results could be relevant, since many PLWH with comorbidities may be candidate to receive RIC regimens, which are characterized by minimal gastrointestinal toxicity, thereby allowing continuation of ART without interruptions [79]. In summary, despite it is currently accepted that alloHSCT in the ART era could be feasible and effective in PLWH affected with hematological malignancies, including AML, due to the limited number of patients so far reported, no definitive conclusion can be drawn on the best strategies regarding the choice of optimal donor, HSC source, conditioning regimens, and GVHD prophylaxis [80].

Another intriguing challenge could be the possibility to use alloHSCT, regardless of underlying hematological malignancy, as a therapeutic strategy to eradicate HIV infection [78,79]. Even in the absence of a detectable viral load after engraftment during ART, donor-derived CD8+ T cell responses against HIV epitopes can be generated after alloHSCT, suggesting the possibility of a graft-versus-HIV effect [79,87]. However, the eradication of HIV viral reservoir is more challenging because latent virus may persist in several tissues, including lymph nodes, gut and central nervous system [79]. Of particular interest, the possible presence in HSC donors of naturally occurring CCR5 delta 32 homozygous mutation, a 32-base pair deletion which prevents CCR5 coreceptor expression and function, rendering cells resistant to HIV infection, could overcome the issue of donor HSC reinfection [79,80]. Indeed, Hutter et al. reported, a decade ago, the HIV eradication and sustained remission without ART, in the “Berlin patient”, an individual who underwent two alloHSCT procedures using HSC from a donor with CCR5 delta 32 homozygous mutation to treat relapsed AML [89]. Similarly, Gupta et al. recently showed a HIV remission in an adult patient with Hodgkin lymphoma, who received a single alloHSCT using cells from a donor with CCR5 delta 32 homozygous mutation [96]. This latter case demonstrated that the “Berlin patient” could not be an anecdotal anomaly. Moreover, it may be argued that the remission of HIV infection can also be obtained with reduced intensity drug regimens, the total body irradiation may not be mandatory and a single alloHSCT may be sufficient to eradicate HIV infection [96]. However, it should be noted that homozygous mutation of CCR5 delta 32 is found in only around 1%–3% of northern European subjects, whereas its prevalence is lower in other population groups, namely African and Asian people, resulting in a very low likelihood to identify a potential HLA-matched unrelated donor with such genetic features [79,80]. The prospective BMT CTN-0903/AMC-080 clinical trial allowed for search to identify potential HSC donors with homozygous mutation of CCR5 delta 32, but only one patient had such a suitable donor identified. This subject unfortunately experienced a leukemia relapse, precluding any long-term evaluation of the impact of alloHSCT on HIV reservoir [82]. The development of HIV cure strategies based on transplantation of hematopoietic cells in which *CCR5* gene is artificially disrupted, leading to prevention of CCR5 corepressor expression, may be a valid alternative approach for rendering cells resistant to HIV infection [96,97]. Relevant to this, Xu et al. recently described, in a HIV-infected patient with acute lymphoblastic leukemia, the proof of principle that transplantation and long-term engraftment of CRISP-edited *CCR5*-ablated allogeneic HSC can be obtained, without significant adverse events [98]. However, the percentage of CCR5 disruption in lymphocytes was only approximately 5%, indicating a low efficiency of CCR5 targeting, which was not adequate to achieve the complete eradication of HIV infection [98]. Therefore, further investigations on gene-editing strategies to target HIV infection are warranted [97,98].

## 8. Conclusions

Even if the incidence of AML in PLWH has not actually been precisely defined, it is generally considered an uncommon finding. However, with the widespread use of ART, life expectancy of newly infected HIV-positive patients is estimated to rival that of age-matched HIV-negative individuals, so that the occurrence of AML is expected to progressively increase, leading to increase in morbidity and mortality in this population [22]. Despite intensive chemotherapeutic approaches including alloHSCT procedures are currently considered feasible and relatively safe, with a multidisciplinary approach, in selected fit patients with AML and well-controlled HIV infection by ART, survival outcomes are still generally unsatisfactory, raising the need to improve both prognostic stratification and treatment of AML [22,36,37,38,39,48,53,54,79,80,82]. In conclusion, several controversial questions about the management of AML in PLWH could be raised, but fewer evidence-based answers could so far be provided, due to the limited number of cases reported in the literature, mainly as case reports or small retrospective case series [22]. Prospective multicenter clinical studies are warranted to more precisely define epidemiology and cytogenetic/molecular features of AML in PLWH, but also to standardize and improve its therapeutic management.

## Figures and Tables

**Table 1:** Acute myeloid leukemia (AML) and HIV infection: demographics and patients’ characteristics at diagnosis.

Reference/n° of cases	Data Collection Period	Median Age (Range), Years	Sex (M/F)	Median HIV Infection Duration	Anti-Retroviral Therapy	Median CD4+ T Cell Count (cells/μL)	Median HIV RNA Viral Load (copies/mL)
Aboulafia et al., 2002 [39]/47 cases *	1986–2001	38(18–70)	39/8	48 months (range, 7–180)	Information NA for most patients	210 (range, 5–1200)	Available in only 5 patients (range, <400–171,000)
Mani et al., 2009 [48]/ 5 t-MN cases ^	1998–2008	39(33–54)	5/0	9 years (range, 2–11)	Yes in 3 cases. NA for 2 patients.	224 and 600 in two patients at diagnosis. NA in 3 cases	Undetectable in two patients, 571 at time of 2^nd^ relapse in another case. NA for 2 patients.
Evans et al., 2012 [32]/9 cases + 22 previously published cases with available karyotype **	2002–2010	44(30–62) for 9 newly identified cases	8/1	range, 0–10 years (among 8 newly diagnosed cases)	NA	153 (<200 in 19 patients) among all 31 cases	Available only for the eight newly identified patients. 103 (range, <40–42,270)
Dy et al., 2012 [52]/5 cases + 68 previously reported cases ^^	1986–2011	40(7–70)	NA	5 years (range, 0.25–28)	NA	NA	NA
Hagiwara et al., 2013 [36]/13 cases ***	1991–2010 (Japanese national survey)	42(21–70)	44/3	28 months (range, 0–204)	68.1% patients received ART before diagnosis	255 (range, 1–1371)	55
Kaner et al., 2015, 2016 [38,53]/9 cases	1997–2016 (retrospective)	56(NA)	NA	13.7 years	All patients received ART at time of AML diagnosis	340 (<200 in 4 cases)	NA
Rabian et al., 2017 [37]/42 cases	2000–2016 (retrospective)	50(46–58)	31/11	NA	NA	347 (range, 210–571)	0(range, 0–2.17)
Cattaneo et al., 2019 [54]/15 cases ^^^	1994–2019 (retrospective)	49(28–67)	17/6	115 months (range, 0–396)	Yes	336 (range, 50–2048)	NA

HIV, human immunodeficiency virus; n°, number; NA, not available; t-MN, therapy-related myeloid neoplasm; ART, anti-retroviral therapy; * The analysis included 5 newly identified patients and a review of 42 additional cases previously reported in the literature; ^ The manuscript showed a total of 5 patients with t-MN, including 4 previously described cases, as shown in Aboulafia et al. [39]; ** The analysis of previously reported patients was limited to cases with available cytogenetic data and CD4+ T cell counts. ^^ The current review of the literature included cases previously summarized in Aboulafia e al. [39] and Evans et al. [32]; *** Patients’ characteristics referred to the entire cohort of 47 patients with hematologic malignancies, including 13 AML cases; ^^^ Patients’ characteristics referred to the whole cohort of 23 patients with acute leukemia or high-risk myelodysplastic syndrome (MDS), including 15 AML cases.

**Table 2:** Clinical features and treatment outcomes of acute myeloid leukemia (AML) cases in HIV+ patients.

Reference	FAB/WHO AML Classification	Median WBC Count at AML Diagnosis (×10^9^/L)	Cytogenetic/Molecular Features	Anti-Leukemic Treatment	HSCT	CR after Induction Therapy/Survival Outcomes	Infectious and Non-Infectious Complications
Aboulafia et al., 2002 [39]	M2 and M4 subtypes in 29/45 cases (64%)	14(range, 0.6–256)	Cytogenetics available in 16 patients (NK in 3 cases, abnormalities in chromosome 7 in six patients)	Standard remission induction treatment for 29 patients	Autologous HSCT in 3 cases.	CR in 26 of 29 patients (83%)/Median OS 7.5 months in chemotherapy-treated cases, compared with median OS 1 month (range, 2 days–3 months) in patients receiving BSC	Five toxic deaths after induction chemotherapy (17.2%)
Mani et al., 2009 [48]	t-MN (5 cases t-AML)	17(range, 0.2–154)	Adverse risk karyotype in 4 cases.	Remission induction chemotherapy	-	CR in 1 case/Median OS 4 weeks (range, 2–16 weeks)	Early death for infectious complications in 4 cases
Evans et al., 2012 [32]	M2 (29.2%) M4-M5 (45.8%)	NA	Favorable, intermediate, adverse risk karyotype in 6 (19%), 16 (52%), 9 (29%) cases, respectively/*FLT3* WT in 6 newly identified cases with available molecular analysis	Overall, 24 patients received intensive remission induction therapy	-	CR in 19/24 cases (79.1%)/Median OS 10.5 months (10.5, 13.5, 5 months for cases with favorable, intermediate and adverse risk cytogenetics, respectively)	Death due to infection in 6 cases
Dy et al., 2012 [52]	M2 (22.6% of cases), M4 (22.6% of cases)	NA	Of 26 patients with available karyotype, 7 favorable, 7 intermediate, 12 unfavorable	Most patients (46) received standard intensive induction therapy	NA	CR in 33/46 cases (71.7%)/Median OS 1 month and 13.2 months for untreated and treated patients, respectively	NA
Hagiwara et al., 2013 [36]	M1, M2, M3, M4, M5 subtypes identified	NA	NK 4 cases, CBF 2 cases, t(15;17) 1 case, CK 3 cases	Most patients received standard chemotherapy	2 cases (15.4%)	70%/Median OS 13 months	NA
Kaner et al., 2015, 2016 [38,53]	NA (t-MN in 1 case)	NA	NA	NA	NA	NA/Median OS 9.5 months	NA
Rabian et al., 2017 [37]	NA (secondary AML in 17 cases, 40.5%)	NA(hyperleukocytosis in 7 cases, 16.7%)	Unfavorable cytogenetic risk in 14 cases, (37.8%)	Intensive chemotherapy in 27 cases (64.3%). Moderate intensity treatment in 6 cases (14.3%)	7 cases (16.7%)	CR in 24 of 42 cases (57.1%)/2-year OS 29%	NA
Cattaneo et al., 2019 * [56]	NA	NA	Six of 20 evaluable cases (30%) had adverse cytogenetics. Inv(16) in one case. *NPM1* mutation in 2 cases. *FLT3* and *TP53* mutations in one case each.	Intensive chemotherapy in 17 cases. BSC in 2 AML cases.	Autologous 3 cases, allogeneic 7 cases (9 AML, 1 MDS)	CR in 11 of 17 patients (65%). After second induction CR 76.5%. After 21 months follow-up, median OS 17 months and 2-year OS 41.2%	Two of 17 patients (12%) died during induction (1 PJP, 1 septic shock).

FAB, French-American-British classification; WHO, World Health Organization classification; WBC, white blood cell; HSCT, hematopoietic stem cell transplant; CR, complete remission; NK, normal karyotype; OS, overall survival; BSC, best supportive care; t-MN, therapy-related myeloid neoplasm; NA, not available; WT, wild type; CBF, core-binding factor; CK, complex karyotype; MDS, myelodysplastic syndrome; PJP, Pneunocystis jirovecii pneumonia. * Clinical data referred to the whole cohort of 23 patients with acute leukemia or high-risk MDS, including 15 AML cases.

**Table 3:** Acute promyelocytic leukemia (APL) in patients with HIV infection: review of detailed cases reported in the literature.

Reference	Age (years)/Sex	WBC/Plt Counts (×10^9^/L)	APL Risk Group	Time Elapsed between HIV Detection and APL Diagnosis	CD4+ T Cell Count (cells/μL)/HIV RNA (copies/mL)	Concurrent ART	Remission Induction Therapy	Response to Induction Treatment	Consolidation	Maintenance	Survival Outcome/Follow- Up*
Calvo et al., 1997 [67]	30/M	4.8/2	Intermediate	2 years	240/NA	NA	ATRA alone	Morphologic CR at day 29, therefore molecular	Daunorubicin, cytarabine/mitoxantrone, cytarabine	NA	Alive/8 months
Gatphoh et al., 2001 [68]	22/F	16/30	High	NA	NA/NA	NA	NA	Refractory	NA	NA	NA/NA
Sutton et al., 2001 [35]	36/M	4/NA	Low or intermediate	Concurrent detection	400/NA	NA	ATRA alone	Morphologic CR, but relapse after 303 days	NA	MTX, 6-MP	Dead/350 days
Kudva et al., 2004 [69]	27/M	8/19	Intermediate	8 years	356/undetectable	Indinavir (switched to nelfinavir), lamivudine, zidovudine (switched to stavudine)	ATRA, idarubicin, cytarabine	Morphologic CR at day 30; cytogenetic and molecular CR at week 9	High-dose cytarabine	ATRA, MTX, 6-MP	Alive/40 months
De Vita et al., 2006 [70]	46/F	5.1/1	Intermediate	2 years	>500/<50	Efavirenz, tenofovir, lamivudine	ATRA, idarubicin	Molecular CR	ATRA, idarubicin/ATRA, mitoxantrone	ATRA, MTX, 6-MP	Alive/21 months
Boban et al., 2009 [71]	35/M	1.6/28	Intermediate	10 years. Previous history of PCNSL in CR after WBRT.	184/<50	Stavudine, lopinavir/ritonavir	ATRA, idarubicin	Morphologic and molecular CR	ATRA	NA	Alive/14 months
Malik et al., 2009 [72]	37/M	1.6/112	Low	7 years	>800/undetectable	Lamivudine, neviropine, didanosine	ATRA, idarubicin	Morphologic and cytogenetic CR at day 77	No	ATRA (scarce compliance because of nausea)	Relapse after 1 year. Salvage treatment with ATO, achieving second CR. Alive/17 months
Drilon et al., 2010 [66]	43/F	40.7/15	High	Concurrent detection	118/>500,000	Atazanavir (switched to fosamprenavir), tenofovir/emtricitabine, raltegravir	ATRA, idarubicin	Morphologic and molecular CR at day 29	ATRA, idarubicin/ATRA, mitoxantrone	ATRA, MTX, 6-MP	Alive/8 months
Kunitomi et al., 2019 [65]	32/M	4/22	Intermediate	5 months	38/75.4	Abacavir/lamivudine, darunavir, ritonavir	ATRA, idarubicin, cytarabine	Morphologic CR at day 40	ATRA, idarubicin/ATRA, mitoxantrone	ATRA, MTX, 6-MP	Alive/38 months
Kunitomi et al., 2019 [65]	46/M	10/19	Intermediate	5 months	264/325	Raltegravir, emtricitabine, tenofovir	ATRA, idarubicin	Morphologic CR at day 31	ATRA, idarubicin/ATRA, mitoxantrone	Not administered because of liver dysfunction	Alive/30 months

WBC, white blood cell; Plt, platelets; ART, anti-retroviral therapy; NA, not available; ATRA, all-trans retinoic acid; CR, complete remission; MTX, Methotrexate; 6-MP, 6-mercaptopurine; PCNSL, primary central nervous system lymphoma; WBRT, whole-brain radiotherapy; ATO, arsenic trioxide; * Last clinical follow-up at time of publication.

**Table 4:** Allogeneic HSCT in patients with acute myeloid leukemia and HIV infection in the ART era.

Reference	n° of AlloHSCT (n° of AML cases)	Age (years)/ Sex (M/F)	CD4+ T Cell Count (cells/μL)/HIV RNA Viral Load (copies/mL) at Time of Transplant	State of Disease at Transplant	Donor Type/ HSC Source	Conditioning Regimens	Neutrophil Engraftment/Platelet Engraftment (days)	GVHD Prophylaxis/ GVHD Incidence	Infectious Complications	Survival Outcomes *
Kang et al., 2002 [83]	2(1)	42/NA	200/494	CR	MSD/PBSC	Non-myeloablative CY/Flu	NA/NA	CyA/grade II acute GVHD, limited chronic GVHD	CMV reactivation	Alive in CR 2 years after transplant
Sorà et al., 2002 [84]	1(1)	33/F	294/undetectable	CR	MSD/PBSC	Myeloablative BuCY	+9/+9	CyA/ grade II acute GVHD; mild limited skin chronic GVHD	Fever of unknown origin	Alive in CR 39 months after HSCT
Shamansky et al., 2004 [85]	1(1)	47/M	NA/ NA	NA	MSD/PBSC	Flu/Melphalan	+16/+16	CyA, MTX/ NA	NA	Alive in CR 250 day after HSCT
Avettand-Fenoel et al., 2007 [86]	1(1)	17/M	NA/undetectable	CR2	MUD /BM	Idarubicin, Flu, Cytarabine	+19/+19	CyA, horse ATG/ Grade III skin and digestive GVHD	Several not specified infections	Death for multivisceral failure on day +191 post HSCT
Woolfrey et al., 2008 [87]	2(2)	case 1 39/M case 2 33/M	case 1 262/<case 2 287/<3030	NA	case 1 MUD/PBSC case 2 MSD/PBSC	Non-myeloablative Flu/TBI	NA	CyA, MMF/ Patient 1 developed skin and gut acute GVHD at immune suppression withdrawal for AML relapse. No GVHD for patient 2.	In patient 1 CMV reactivation, P. aeruginosa sepsis and sinuses mucormycosis, concurrently with BO, leading to death. No infections for patient 2.	Patient 1 died on day +301 after HSCT. Patient 2 alive longer than 180 days after HSCT.
Hamadani et al., 2009 ^ [88]	3(1)	case 1 with AML 39/NA	189/undetectable	CR2	MSD/NA	RIC (Bu/Flu)	NA/NA	MTX, MMF/ acute skin GVHD	CMV reactivation	All three patients alive without evidence of disease relapse at a median follow-up of 375 days
Hutter et al., 2009 [89]	1(1) ”Berlin patient”	40/M	415/undetectable	Relapsed at 1st HSCT. CR2 for 2nd HSCT	1^st^ HSCT MUD/PBSC 2^nd^ HSCT MUD (same donor)/PBSC	1^st^ AMSA, Flu-TBI-cytarabine 2^nd^ TBI, after reinduction with cytarabine and GO	+13	1^st^ rabbit ATG, CyA, MMF 2^nd^ CyA/ Grade I skin GVHD	No serious infections were observed.	Alive in remission at 42 months follow-up.
Oka et al., 2010 [90]	1(1)	39/M	NA/61	CR	MUD/BM	Myeloablative TBI/CY/VP-16	+18/+22	CyA, MTX/acute skin GVHD, chronic extensive GVHD	Febrile neutropenia, CMV reactivation	Alive in CR with undetectable HIV-RNA 21 months after transplant
Polizzotto et al., 2010 ^ [91]	3(2)	case 1 38/M case 2 41/M	case 1 578/undetectable case 2 275/undetectable	case 1 relapse case 2 CR3	case 1 MUD/PBSC case 2 MSD/PBSC	case 1 CY TBI case 2 CY TBI	case 1 day +48 case 2 day +21	CyA/No GVHD in case 1, grade II skin GVHD in case 2	BK virus cystitis and E. coli sepsis in case 1. Febrile neutropenia in case 2	Patient 1 died of renal failure due to glomerulopathy 24 months after HSCT. Patient 2 died of MDR pseudomonal sepsis on day +78.
Hagiwara et al., 2013 [36] **	4 of the entire cohort of 47 cases (2)	NA	NA	NA	NA/NA	NA	NA/NA	NA/one patient died of acute GVHD	NA	Three patients survived longer than 4 years
Mulanovich et al., 2016 ^ [92]	5(2)	case 1 57/M case 4 55/M	case 1 95/<48 case 4 208/<20	CR (both patients)	case 1 MUD/NA case 4 MUD/NA	case 1 RIC Flu-melphalan-ATG case 4 myeloablative Bu-Flu	median +17 (range, 13-19) for all 5 patients	Standard prophylaxis/ case 1 No GVHD, case 4 grade I-II acute GVHD	Febrile neutropenia in patient 1. CMV reactivation in 4 of 5 patients.	Patient 1 and 4 died of relapsed leukemia 6 and 7 months after HSCT, respectively.
Johnston et al., 2016 ^ [93]	13 patients (3 AML) receiving 15 HSCT, namely 7 autologous, 8 allogeneic	case 8 39/M case 9 33/M case 13 54/M Median age of entire cohort 42 (range, 28-60)	case 8 373/<30 case 9 287/<30 case 13 141/ undetectable	NA	case 8 MUD/NA case 9 MSD/NA case 13 MUD/NA	cases 8 and 9 non-myeloablative Flu/TBI case 13 myeloablative Bu/Clofarabine	Data available for the entire 13 patient cohort. Median +16 (range, 10–24)/ Median +15 (range, 9–34)	Tacrolimus combined with MMF or MTX/ Grade II GHVD in patients 8 and 13	CMV reactivation only in patient 8	Four of 13 patients alive, with median OS of the cohort 9.6 months. Patients 8 and 13 died of disease relapse at 9.6 and 9 months, respectively. Patient 9 alive at 7.1 years after transplant.
Rabian et al., 2017 [37] **	8 of the whole cohort of 47 patients (7)	NA	NA/NA	NA	NA/NA	NA	NA/NA	NA/NA	NA	NA
Metha et al., 2018 ^^ [81]	108 (alloHSCT 15.5% cases; leukemia in 8 cases, 7.1%)	mean 44.7/84.2% M	NA/NA	NA	NA/ PBSC in 95.6% of cases	chemotherapy 88% of cases, TBI 7.2% of cases	NA/NA	NA/ NA (no difference in incidence of GVHD between HIV+ and HIV- patients)	In alloHSCT, higher incidence of non-tuberculous mycobacterial and CMV infections compared with HIV- patients	No difference in mortality between HIV+ and HIV- patients. Significant predictors of mortality included bacteremia, opportunistic infections, GVHD, intubation.
Kanellopoulos et al., 2018 [94]	1(1)	39/M	755/ undetectable	CR	MUD/ PBSC	RIC Flu-Melphalan	+15/+13	CyA, Alemtuzumab/ Limited chronic oral GVHD	Early post-HSCT pneumonia	Alive in sustained CR >4 years post transplant
Kwon et al., 2019 ^^ [95]	22(7)	median 44 (range, 30-57)/17 M (77%)	median 192 (range, 25-1699) / HIV RNA detectable in only 2 patients (9.1%)	CR1 9 (41%) CR>1 5 (23%) PR 5 (23%) refractory 3 (13%)	MSD 11 cases, MUD 5 cases, haploidentical 4 cases, CB 2 cases/PBSC in 91% of cases	Myeloablative in 11 cases, non-myeloablative in 11 cases	median 14.5 days (range, 11-22)/median 20.5 days (range, 6-480)	Prophylaxis according to donor and graft source (MTX+CyA in 50% of cases). CY post-transplant in haploidentical HSCT/acute GVHD 44%, moderate-severe chronic GVHD 41%	76.2% of patients developed multiple infectious complications, mainly of viral origin. Bacterial infections in 31% of cases.	At a median follow-up of 65 months, OS and EFS 46%. NRM 14% and 31% at 12 and 60 months, respectively.
Ambinder et al., 2019 ^^ [82]	17(9)	median 47 (range, 25-64)/17 M	median 224 (range, 55-833)/ undetectable in 15/17 cases (88.2%)	CR1 in 8 cases CR2 in 3 cases (among 11 patients with AML or ALL)	MSD (4 cases), MUD (9 cases), MMSD (3 cases), MMUD (1 case)/ BM	RIC: Bu-Flu, Flu-Melphalan. Myeloablative: Bu-Flu, CY/TBI	median +17 (range, 11-22)/ median 19 days	Tacrolimus+MTX, tacrolimus+ sirolimus, tacrolimus+MMF + post-transplant CY/ acute GVHD in 7 cases, chronic GVHD in 3 patients	Infectious events occurred in 11 patients (64.7%), for a total number of 55 infections, mainly bacterial.	6-month OS 82.4%, 1-year OS 58.8%, estimated 2-year OS 52.3%. The 1-year rate of disease progression was 29.4%
Cattaneo et al., 2019 *** [54]	7 alloHSCT + 3 autoHSCT among 10 patients (9 AML, 1 MDS)	NA for patients undergoing HSCT	NA/NA for patients undergoing HSCT	First line treatment (CR)	NA	NA	NA	NA/NA	Two deaths after alloHSCT (1 acute GVHD, 1 infection). One death after autoHSCT for hemorrhagic stroke	NA

HSCT, hematopoietic stem cell transplant; ART, anti-retroviral therapy; n°, number; AML, acute myeloid leukemia; HSC, hematopoietic stem cells;GVHD, graft versus host disease; NA, not available; CR, complete remission; MSD, matched sibling donor; PBSC, peripheral blood stem cells; CY, cyclophosphamide; Flu, fludarabine, CyA, cyclosporine; CMV, cytomegalovirus; Bu, busulfan; MTX, methotrexate; MUD; matched unrelated donor; ATG, anti-thymocyte globulin; TBI, total-body irradiation; MMF, mycophenolate mofetil; BO, bronchiolitis obliterans; RIC, reduced intensity conditioning; AMSA, amsacrin; GO, gemtuzumab-ozogamycin; VP-16, etoposide; MDR, multi-drug resistant; PR, partial remission; CB, cord blood; OS, overall survival; EFS, event-free survival; NRM, non-relapse mortality; ALL, acute lymphoblastic leukemia; MMSD, single antigen mismatched sibling donor; MMUD, single antigen mismatched unrelated donor; MDS, myelodysplatic syndrome. * Last clinical follow-up at time of publication. ^ Detailed information in this table is mainly referred to patients affected with AML ** Demographics referred to the entire cohort, whereas no specific information is provided for the patients undergoing HSCT. ^^ Demographics and clinical outcomes referred to the whole cohort of patients with hematologic malignancies receiving HSCT. *** Clinical data referred to the whole cohort of 23 patients with acute leukemia or high-risk MDS.
